# Supplementary material for: IGF2BP3 promotes mRNA degradation through internal m7G modification
Source: Nat Commun. 2024 Aug 28;15:7421. doi: 10.1038/s41467-024-51634-w (PMC11358264; doi:10.1038/s41467-024-51634-w)
Supplement: Supplementary file 1 — Supplementary Information [file 41467_2024_51634_MOESM1_ESM.pdf]

Supporting Information

IGF2BP3 promotes mRNA degradation through internal m^7G modification

Chang Liu[1,2,5,8], Xiaoyang Dou[1,2,8], Yutao Zhao[1,2,8], Linda Zhang[1,2], Lisheng Zhang[1,2,6,7], Qing Dai[1,2], Jun Liu[3,4], Tong Wu[1,2], Yu Xiao[1,2], and Chuan He[1,2,*]

[1]Department of Chemistry, Department of Biochemistry and Molecular Biology, and Institute for Biophysical Dynamics, The University of Chicago, Chicago, IL 60637, USA

[2]Howard Hughes Medical Institute, The University of Chicago, Chicago, IL 60637, USA

[3]State Key Laboratory of Protein and Plant Gene Research, School of Life Sciences, Peking-Tsinghua Center for Life Sciences, Peking University, Beijing 100871, China

[4]Beijing Advanced Center of RNA Biology (BEACON), Peking University, Beijing 100871, China

[5]Present address: Department of Genetics, School of Medicine, Stanford University, Stanford, CA 94305, USA

[6]Present address: Division of Life Science, The Hong Kong University of Science and Technology, Hong Kong, China

[7]Present address: Department of Chemistry, The Hong Kong University of Science and Technology, Hong Kong, China

[8]These authors contributed equally: Chang Liu, Xiaoyang Dou, Yutao Zhao

[*]Correspondence authors: Chuan He (chuanhe@uchicago.edu)

This file contains:

Supporting Figures 1-10

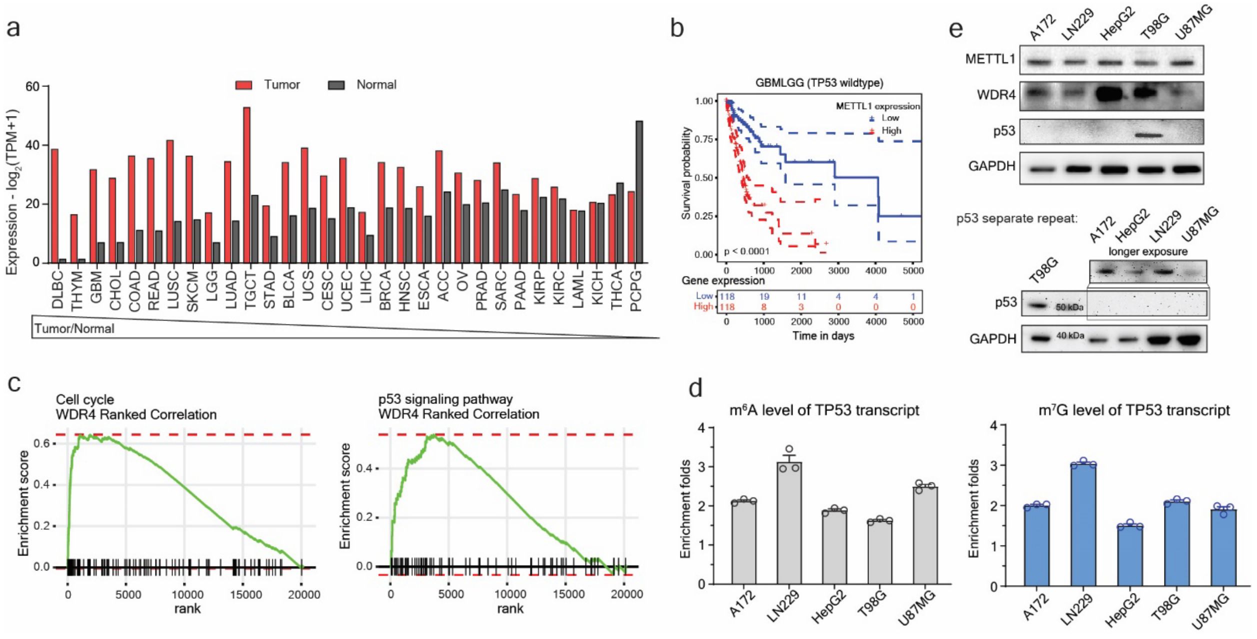

Supplementary Fig. 1: METTL1 and WDR4 are involved in glioma and p53 related pathways.

a Expression level of *METTL1* in tumors and normal tissues across TCGA cancer types, ranked with the ratio of the expression in tumor divided by the expression in normal tissues. **b** Kaplan-Meier survival analysis in TCGA database for Glioma (TCGA-GBMLGG) cases with wildtype TP53. The patients were divided into two groups of equal size based on *IGF2BP3* levels. *P* value was detected by the log-rank test. **c** Gene Set Enrichment Analysis (GSEA) of genes in ‘Cell cycle’ and ‘p53 signaling pathway’ KEGG pathways against ranked list of genes according to *WDR4* expression level. **d** Relative m^6A (left) and m^7G (right) methylation levels of *TP53* transcript. Mean ± SEM of three independent experiments. **e** Western blot showing relative quantity of METTL1, WDR4, and TP53 proteins in cell lines. This experiment was repeated independently twice with similar results. All source data are provided as a Source Data file.

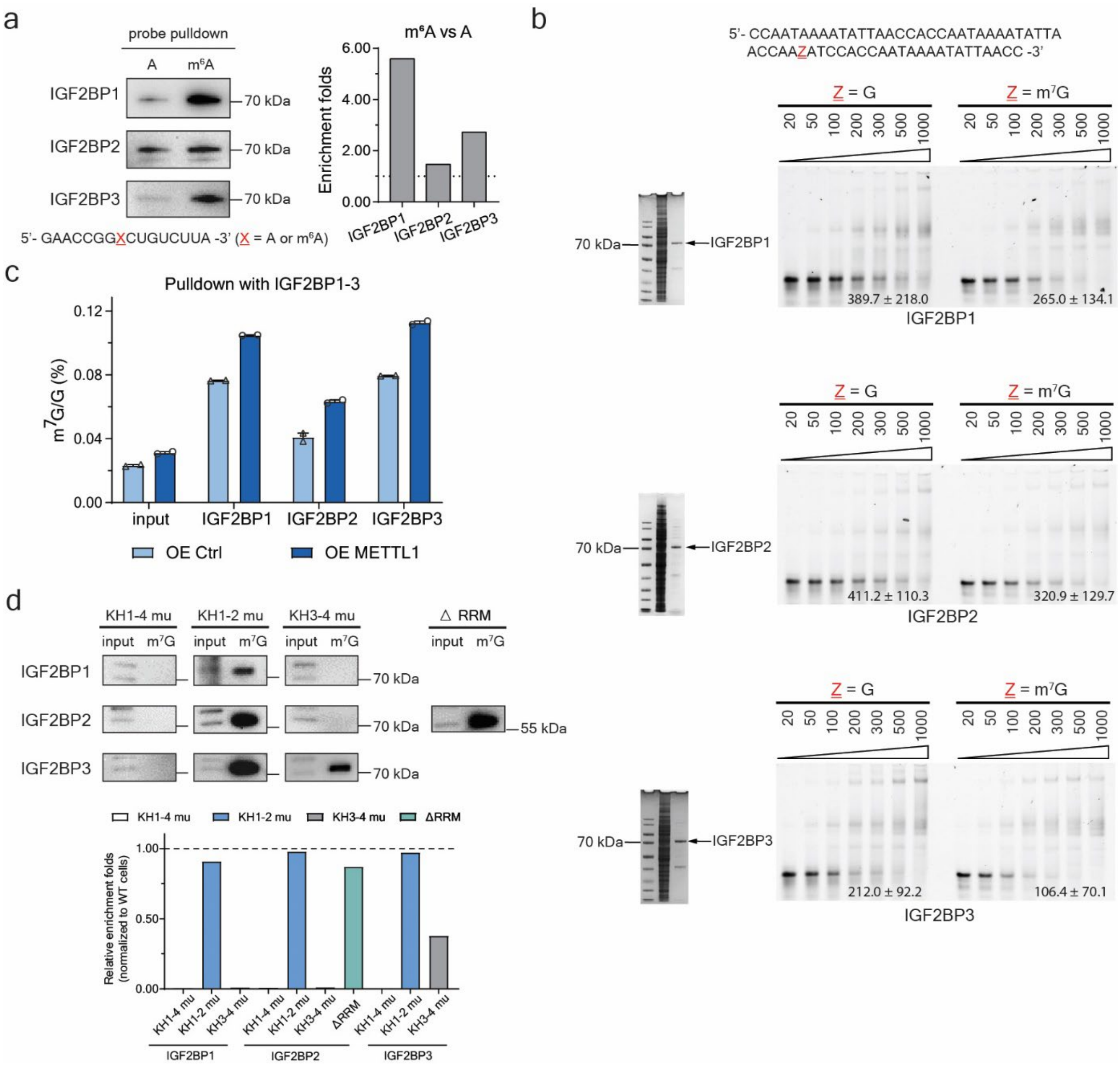

Supplementary Fig. 2: Binding of IGF2BP family proteins to internal m^{7}G and m^{6}A in RNA.

a Western blot of HepG2 cell lysate *in vitro* pulldown with m^{6}A probe and A probe, the estimation of enrichment folds is plotted on the right. This experiment was repeated independently twice with similar results. The probe sequence is listed below the blots. **b** Gel-shift assay demonstrating the binding of FLAG-tagged IGF2BP family proteins (from up to down, IGF2BP1, IGF2BP2, IGF2BP3) with m^{7}G-methylated and unmethylated RNA probes. 4 nmol RNA probe was labelled with Cy3 and the protein concentration ranged from 20 nM to 1 μM. (K_d, nM, indicated at the bottom right of each gel). The probe sequence is listed at the top. This experiment was repeated independently twice with similar results. **c** LC-MS/MS quantification of m^{7}G/G levels showing enrichment of m^{7}G after immunoprecipitation using IGF2BPs antibodies in cells with METTL1 overexpression compared to control. Mean ± SEM of two independent experiments. **d** Methylated RNA probe pulldown followed by western blotting showed *in vitro* binding of the baits with KH domain-mutated IGF2BP variants and RRM domain-depleted IGF2BP2. Top: western blotting showing the enrichment of m^{7}G probes with each variant. Bottom: quantification of relative enrichment of m^{7}G probes compared to those in wild-type cell lysates (Fig. **2b**). KH1-2, KH3-4, and KH1-

4, refer to mutation of GxxG to GEEG in corresponding KH domains in the full-length proteins. This experiment was repeated independently twice with similar results. All source data are provided as a Source Data file.

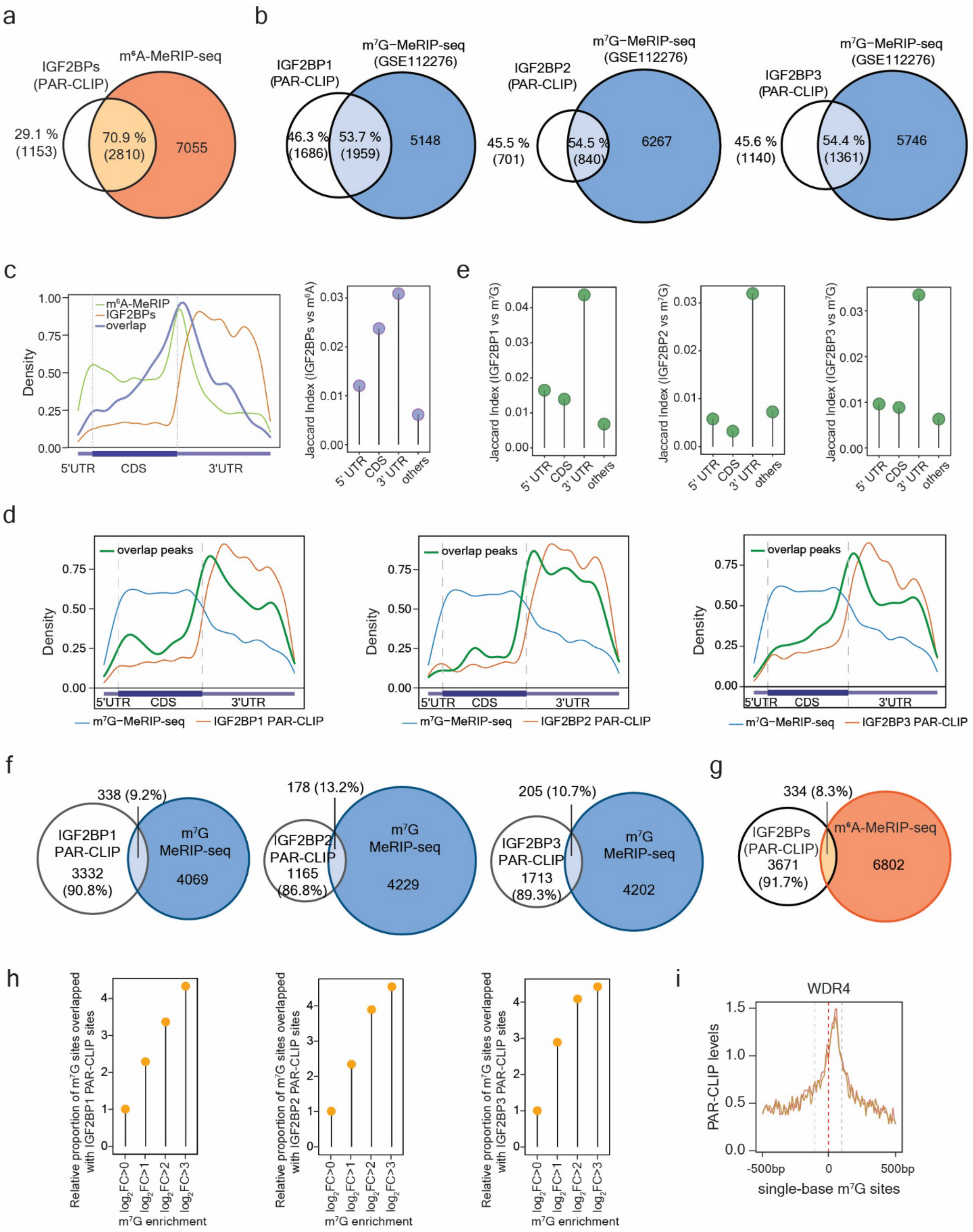

Supplementary Fig. 3: Transcriptome binding profile of IGF2BP family proteins to internal m^7G.

a Venn diagram of overlaps of transcripts that were bound by IGF2BPs and modified with m^6A. **b** Venn diagram of overlaps of transcripts that were modified with m^7G and bound by IGF2BP1 (left), IGF2BP2 (middle) and IGF2BP3 (right). **c** Left: metagene plot of IGF2BPs binding sites, m^6A modified sites and their overlapping sites. Right: Lollipop plot showing the overlaps between m^6A and IGF2BPs quantified using Jaccard Index. **d** Metagene plot of IGF2BPs binding sites, m^7G modified sites and their overlapped sites: IGF2BP1 (left), IGF2BP2 (middle), and IGF2BP3 (right). **e** Lollipop plot showing the overlaps between m^7G and IGF2BPs quantified using Jaccard Index: IGF2BP1 (left), IGF2BP2 (middle), and IGF2BP3 (right). **f** Venn diagram of peaks of transcripts bound by IGF2BP1 (left), IGF2BP2 (middle), and IGF2BP3 (right) in the 3'UTR. **g** Venn diagram of overlaps of peaks that were bound by IGF2BPs and modified with m^6A. **h** Proportion of m^7G sites overlapped with IGF2BPs binding sites. m^7G sites were categorized into groups based on m^7G enrichment (log_2FC(IP/Input)): IGF2BP1 (left), IGF2BP2 (middle), and IGF2BP3 (right). **i** Binding intensity of WDR4 at m^7G sites and their flanking 500bp.

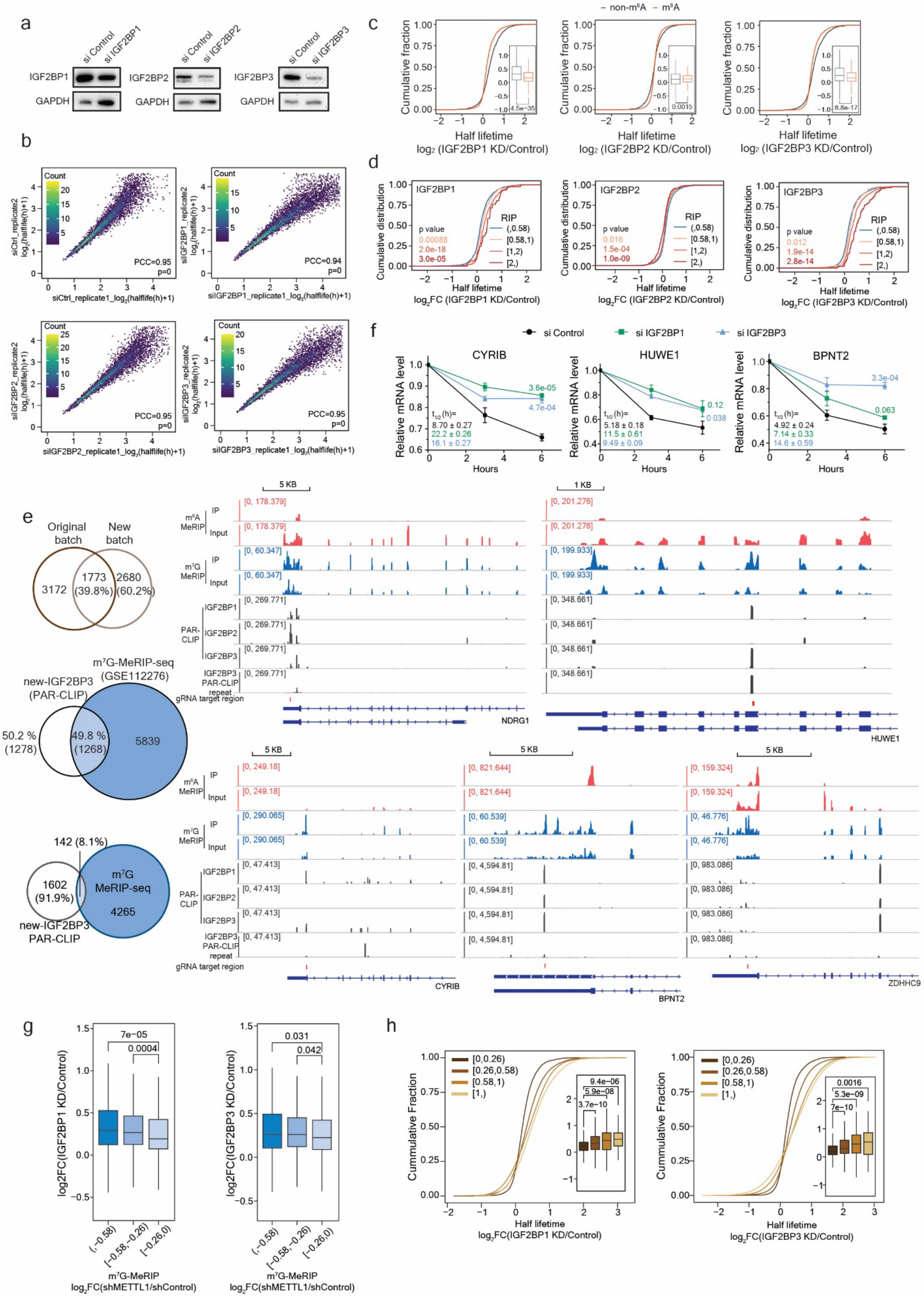
a
si Control
si IGF2BP1
si IGF2BP2
si IGF2BP3
IGF2BP1
IGF2BP2
IGF2BP3
GAPDH
b
Count
PCC=0.95
PCC=0.94
p=0
siCtrl_replicate1_log2(halflife(h)+1)
siCtrl_replicate2 log2(halflife(h)+1)
siIGF2BP1_replicate1_log2(halflife(h)+1)
siIGF2BP1_replicate2 log2(halflife(h)+1)
siIGF2BP2_replicate1_log2(halflife(h)+1)
siIGF2BP2_replicate2 log2(halflife(h)+1)
siIGF2BP3_replicate1_log2(halflife(h)+1)
siIGF2BP3_replicate2 log2(halflife(h)+1)
c
non-m6A
m6A
Cumulative fraction
4.5e-35
0.0015
8.8e-17
Half lifetime
log2 (IGF2BP1 KD/Control)
log2 (IGF2BP2 KD/Control)
log2 (IGF2BP3 KD/Control)
d
Cumulative distribution
IGF2BP1
IGF2BP2
IGF2BP3
p value
RIP
(,0.58)
[0.58,1)
[1,2)
[2,)
0.00088
2.0e-18
3.0e-05
0.016
1.5e-04
1.0e-09
0.012
1.9e-14
2.8e-14
Half lifetime
log2FC (IGF2BP1 KD/Control)
log2FC (IGF2BP2 KD/Control)
log2FC (IGF2BP3 KD/Control)
f
si Control
si IGF2BP1
si IGF2BP3
CYRIB
HUWE1
BPNT2
Relative mRNA level
Hours
3.6e-05
4.7e-04
0.12
0.038
3.3e-04
0.063
t1/2(h)=
8.70 ± 0.27
22.2 ± 0.26
16.1 ± 0.27
5.18 ± 0.18
11.5 ± 0.61
9.49 ± 0.09
4.92 ± 0.24
7.14 ± 0.33
14.6 ± 0.59
e
Original batch
New batch
3172
1773 (39.8%)
2680 (60.2%)
m7G-MeRIP-seq (GSE112276)
new-IGF2BP3 (PAR-CLIP)
50.2 % (1278)
49.8 % (1268)
5839
142 (8.1%)
1602 (91.9%)
m7G MeRIP-seq
4265
new-IGF2BP3 PAR-CLIP
5 KB
1 KB
m6A MeRIP
m7G MeRIP
IP
Input
PAR-CLIP
IGF2BP3 PAR-CLIP repeat
gRNA target region
NDRG1
HUWE1
CYRIB
BPNT2
ZDHHC9
g
7e-05
0.0004
0.031
0.042
log2FC(IGF2BP1 KD/Control)
log2FC(IGF2BP3 KD/Control)
(-,-0.58)
[-0.58,-0.26)
[-0.26,0)
m7G-MeRIP
log2FC(shMETTL1/shControl)
h
[0,0.26)
[0.26,0.58)
[0.58,1)
[1,)
Cummulative Fraction
9.4e-06
5.9e-08
3.7e-10
0.0016
5.3e-09
7e-10
Half lifetime
log2FC(IGF2BP1 KD/Control)
log2FC(IGF2BP3 KD/Control)

Supplementary Fig. 4: IGF2BP1 and IGF2BP3 promote decay of m^7G-modified target transcripts, including METTL1 targets.

a Western blot of *IGF2BP1-3* knockdown in HepG2 cells. This experiment was repeated independently twice with similar results. **b** Correlation analyses for each pair of replicates in the half lifetime decay assay. **c** Cumulative curve of gene half lifetime fold changes (log_2FC) upon knocking down *IGF2BP1* (left), *IGF2BP2* (middle), and *IGF2BP3* (right). Genes were categorized into two groups according to whether they were marked with m^6A or not (non-m^6A). **d** Cumulative curve of gene half lifetime fold changes (log_2FC) upon *IGF2BP1* (left), *IGF2BP2* (middle), and *IGF2BP3* (right) knockdown. m^7G modified genes were categorized into four groups according to IGF2BP1, IGF2BP2, or IGF2BP3 binding intensity calculated by quantifying the log_2 ratio of enrichment in immunoprecipitated (IP) samples compared to input samples, based on the RNA immunoprecipitation (RIP)-seq data, denoted as log_2(IP/Input) respectively. **e** Left: Venn diagrams presenting the consistent and comparable results of the newly repeat batch for PAR-CLIP of IGF2BP3 compared to the original batch. Top: overlaps of transcripts that were bound by IGF2BP3 in the original batch and in the new repeat batch. Middle: overlaps of transcripts that were bound by IGF2BP3 in the new repeat batch and modified with m^7G. Bottom: overlaps of peaks that were bound by IGF2BP3 and modified with m^7G in the 3' UTR in the new repeat batch. Right: IGV plots demonstrating the representative genes with m^7G modification at the loci, bound by IGF2BP proteins, but with little m^6A modification nearby. Y-axis showing counts per ten million reads. **f** Changes in mRNA levels in HepG2 cells with transient knockdown of *IGF2BP1* and *IGF2BP3*. Mean ± SEM of four independent experiments. Two-tailed Student's t-tests were used. *P* values are marked next to the dots. Calculated half lifetimes are marked in the corresponding colors. **g** Gene half lifetime log_2FC upon *IGF2BP1* (left) and *IGF2BP3* (right) knockdown. Hypo-methylated (m^7G) genes by *METTL1* knockdown were further categorized into three groups based on their methylation log_2FC upon *METTL1* knockdown. **h** Cumulative curve of gene half lifetime fold changes (log_2FC) upon *IGF2BP1* (left) and *IGF2BP3* (right) knockdown. The stabilized genes upon *METTL1* knockdown were further categorized into four groups according to their half lifetime log_2FC level comparing *METTL1* knockdown with controls. All source data are provided as a Source Data file.

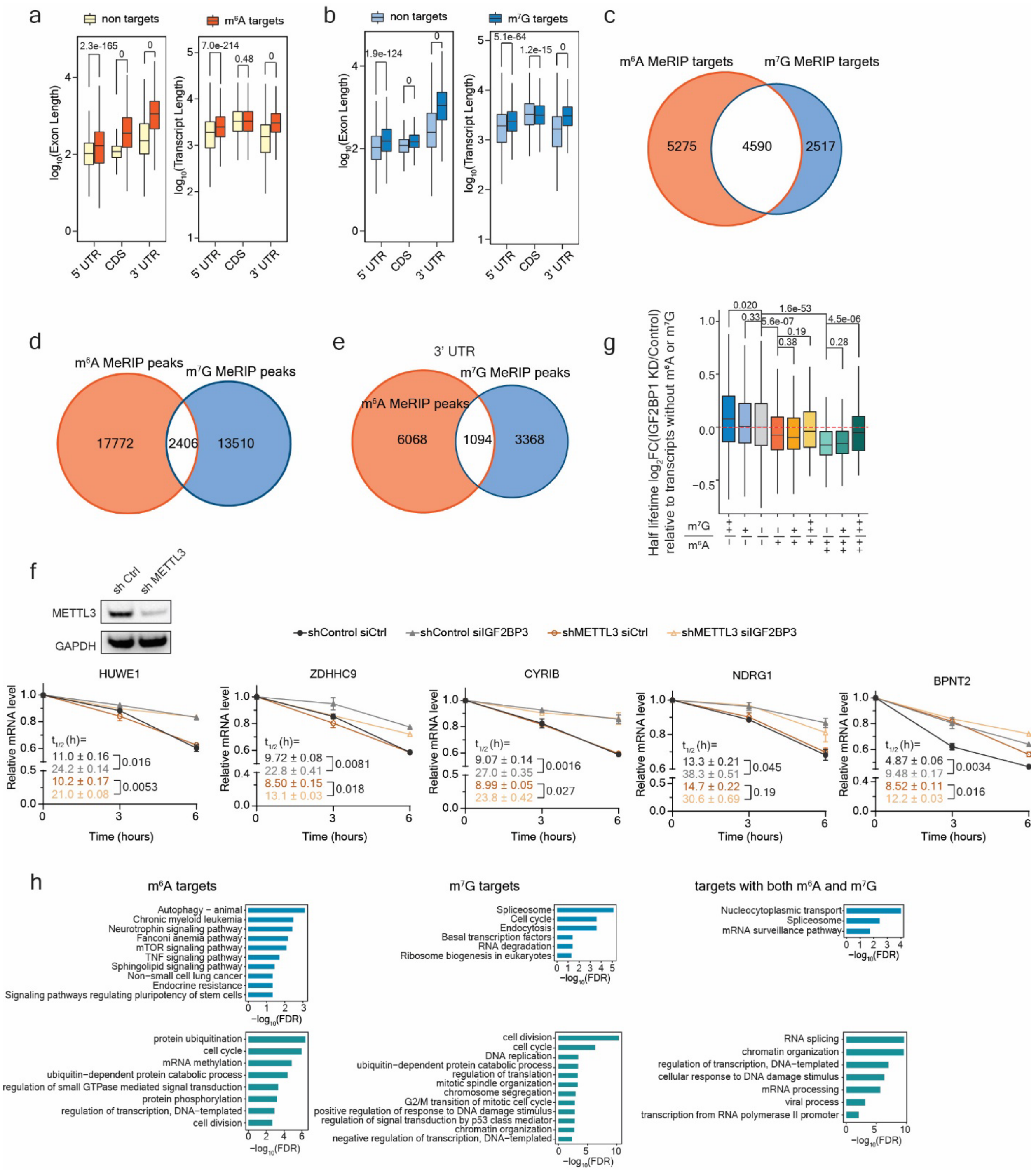

Supplementary Fig. 5: Limited overlaps between m^{6}A- and m^{7}G-marked transcripts when regulated by IGF2BPs.

a Boxplot showing exon length and transcript length. Genes were categorized into two groups according to whether they were marked with m^{6}A or not (non-m^{6}A). **b** Boxplot showing exon length and transcript length. Genes were categorized into two groups according to whether they were marked with m^{7}G or not (non-m^{7}G). **c** Venn diagram of the overlaps of transcripts that were modified with m^{6}A and m^{7}G. **d** Venn diagram of the overlaps of m^{6}A peaks and m^{7}G peaks. **e** Venn diagram of the overlaps of m^{6}A peaks and m^{7}G peaks

in the 3' UTR region. **f** Evaluation of the effect of m^6A writer METTL3 in the regulation of IGF2BP3 on m^7G targets. Top left: western blotting results confirming the stable knockdown of *METTL3* compared to control. This experiment was repeated independently twice with similar results. Bottom right: Changes in mRNA levels in HepG2 cells with transient knockdown of *IGF2BP1* (left), *IGF2BP2* (middle), and *IGF2BP3* (right) in sh*METTL3* HepG2. Mean ± SEM of two independent experiments. Two-tailed Student's t-tests were used. Calculated half lifetimes are marked in the corresponding colors. *P* values are marked next to the pair of calculated half lifetimes. **g** Boxplot showing genes half lifetime fold changes (log_2FC) upon *IGF2BP1* knockdown. Genes were categorized into different groups according to their m^7G or m^6A modification level. '-' represents for $log_2FC(IP/Input) < 0$; '+' represents for $log_2FC(IP/Input) > 0$ and $log_2FC(IP/Input) < 1$; '++' represents for $log_2FC(IP/Input) > 1$. **h** Representative KEGG pathways (top in blue) and GO terms (bottom in green) enriched at targets that are only m^6A modified (left), only m^7G modified (middle), and modified by both (right). All source data are provided as a Source Data file.

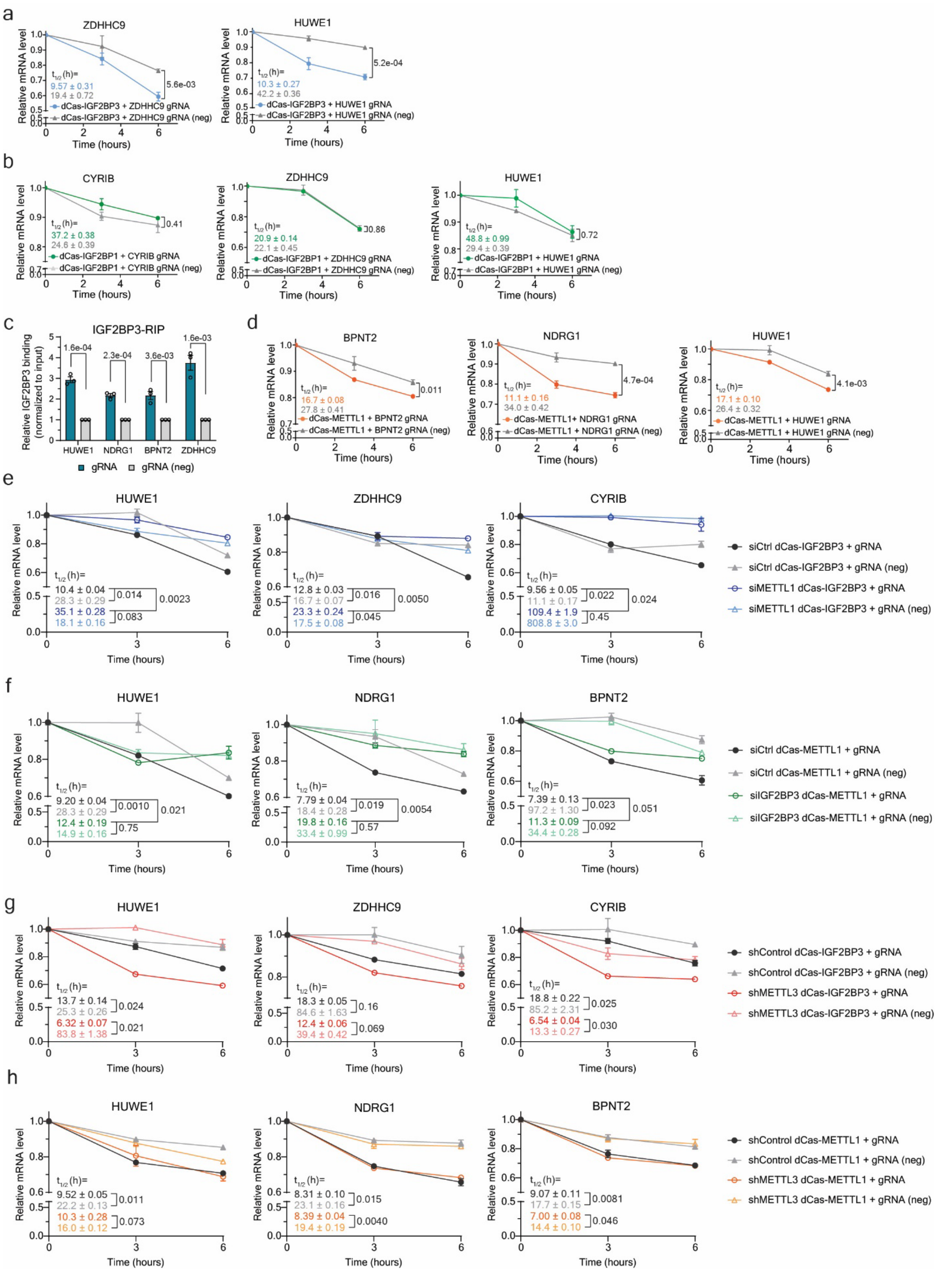
a
ZDHHC9
Relative mRNA level
t1/2 (h)=
9.57 ± 0.31
19.4 ± 0.72
5.6e-03
dCas-IGF2BP3 + ZDHHC9 gRNA
dCas-IGF2BP3 + ZDHHC9 gRNA (neg)
Time (hours)
HUWE1
10.3 ± 0.27
42.2 ± 0.36
5.2e-04
dCas-IGF2BP3 + HUWE1 gRNA
dCas-IGF2BP3 + HUWE1 gRNA (neg)
b
CYRIB
37.2 ± 0.38
24.6 ± 0.39
0.41
dCas-IGF2BP1 + CYRIB gRNA
dCas-IGF2BP1 + CYRIB gRNA (neg)
ZDHHC9
20.9 ± 0.14
22.1 ± 0.45
0.86
dCas-IGF2BP1 + ZDHHC9 gRNA
dCas-IGF2BP1 + ZDHHC9 gRNA (neg)
HUWE1
48.8 ± 0.99
29.4 ± 0.39
0.72
dCas-IGF2BP1 + HUWE1 gRNA
dCas-IGF2BP1 + HUWE1 gRNA (neg)
c
IGF2BP3-RIP
Relative IGF2BP3 binding (normalized to input)
1.6e-04
2.3e-04
3.6e-03
1.6e-03
HUWE1
NDRG1
BPNT2
ZDHHC9
gRNA
gRNA (neg)
d
BPNT2
16.7 ± 0.08
27.8 ± 0.41
0.011
dCas-METTL1 + BPNT2 gRNA
dCas-METTL1 + BPNT2 gRNA (neg)
NDRG1
11.1 ± 0.16
34.0 ± 0.42
4.7e-04
dCas-METTL1+ NDRG1 gRNA
dCas-METTL1 + NDRG1 gRNA (neg)
HUWE1
17.1 ± 0.10
26.4 ± 0.32
4.1e-03
dCas-METTL1 + HUWE1 gRNA
dCas-METTL1 + HUWE1 gRNA (neg)
e
HUWE1
10.4 ± 0.04
28.3 ± 0.29
35.1 ± 0.28
18.1 ± 0.16
0.014
0.083
0.0023
ZDHHC9
12.8 ± 0.03
16.7 ± 0.07
23.3 ± 0.24
17.5 ± 0.08
0.016
0.045
0.0050
CYRIB
9.56 ± 0.05
11.1 ± 0.17
109.4 ± 1.9
808.8 ± 3.0
0.022
0.45
0.024
siCtrl dCas-IGF2BP3 + gRNA
siCtrl dCas-IGF2BP3 + gRNA (neg)
siMETTL1 dCas-IGF2BP3 + gRNA
siMETTL1 dCas-IGF2BP3 + gRNA (neg)
f
HUWE1
9.20 ± 0.04
28.3 ± 0.29
12.4 ± 0.19
14.9 ± 0.16
0.0010
0.75
0.021
NDRG1
7.79 ± 0.04
18.4 ± 0.28
19.8 ± 0.16
33.4 ± 0.99
0.019
0.57
0.0054
BPNT2
7.39 ± 0.13
97.2 ± 1.30
11.3 ± 0.09
34.4 ± 0.28
0.023
0.092
0.051
siCtrl dCas-METTL1 + gRNA
siCtrl dCas-METTL1 + gRNA (neg)
siIGF2BP3 dCas-METTL1 + gRNA
siIGF2BP3 dCas-METTL1 + gRNA (neg)
g
HUWE1
13.7 ± 0.14
25.3 ± 0.26
6.32 ± 0.07
83.8 ± 1.38
0.024
0.021
ZDHHC9
18.3 ± 0.05
84.6 ± 1.63
12.4 ± 0.06
39.4 ± 0.42
0.16
0.069
CYRIB
18.8 ± 0.22
85.2 ± 2.31
6.54 ± 0.04
13.3 ± 0.27
0.025
0.030
shControl dCas-IGF2BP3 + gRNA
shControl dCas-IGF2BP3 + gRNA (neg)
shMETTL3 dCas-IGF2BP3 + gRNA
shMETTL3 dCas-IGF2BP3 + gRNA (neg)
h
HUWE1
9.52 ± 0.05
22.2 ± 0.13
10.3 ± 0.28
16.0 ± 0.12
0.011
0.073
NDRG1
8.31 ± 0.10
23.1 ± 0.16
8.39 ± 0.04
19.4 ± 0.19
0.015
0.0040
BPNT2
9.07 ± 0.11
17.7 ± 0.15
7.00 ± 0.08
14.4 ± 0.10
0.0081
0.046
shControl dCas-METTL1 + gRNA
shControl dCas-METTL1 + gRNA (neg)
shMETTL3 dCas-METTL1 + gRNA
shMETTL3 dCas-METTL1 + gRNA (neg)

Supplementary Fig. 6: Validation with dCas13b-based tethering system.

a-b Changes in mRNA levels in HepG2 cells with introduction of the dCas13b-IGF2BP3 (**a**) and dCas13b-IGF2BP1 (**b**) and guide RNA at the target loci or not (neg). Mean ± SEM of three independent experiments. Two-tailed Student's t-tests were used. *P* values are marked next to the dots. Calculated half lifetimes are marked in the corresponding colors. **c** Changes in IGF2BP3 binding intensity on the representative targets in HepG2 cells with introduction of the dCas13b-METTL1 and guide RNA at the target loci or not (neg). Mean ± SEM of three independent experiments. Two-tailed Student's t-tests were used. *P* values are marked on the top of each group. **d** Changes in mRNA levels in HepG2 cells with introduction of the dCas13b-METTL1 and the guide RNAs at the target loci or not (neg). Mean ± SEM of three independent experiments. Two-tailed Student's t-tests were used. *P* values are marked next to the dots. Calculated half lifetimes are marked in the corresponding colors. **e** Changes in mRNA levels in HepG2 cells under *METTL1* knockdown or control with introduction of dCas13b-IGF2BP3 and the guide RNA at the target loci or not (neg). Mean ± SEM of two independent experiments. Two-tailed Student's t-tests were used. Calculated half lifetimes are marked in the corresponding colors. *P* values are marked next to the pair of half lifetime values. **f** Changes in mRNA levels in HepG2 cells under *IGF2BP3* knockdown or control with introduction of dCas13b-METTL1 and the guide RNA at the target loci or not (neg). Mean ± SEM of two independent experiments. Two-tailed Student's t-tests were used. Calculated half lifetimes are marked in the corresponding colors. *P* values are marked next to the pair of half lifetime values. **g-h** Changes in mRNA levels in HepG2 cells under *METTL3* stable knockdown or control with introduction of the dCas13b-IGF2BP3 (**g**) or dCas13b-METTL1 (**h**) and guide RNA at the target loci or not (neg). Mean ± SEM of two independent experiments. Two-tailed Student's t-tests were used. Calculated half lifetimes are marked in the corresponding colors. *P* values are marked next to the pair of half lifetime values. All source data are provided as a Source Data file.

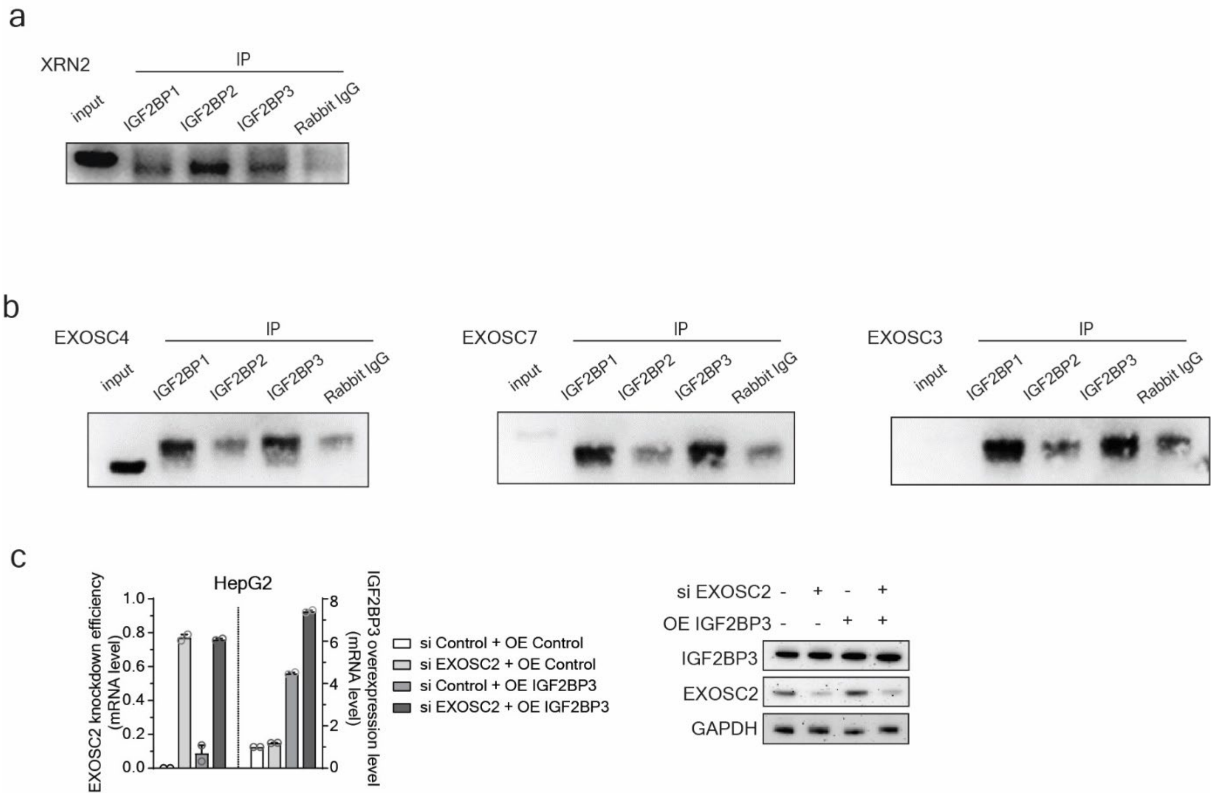

Supplementary Fig. 7: Mechanistic studies of the decay process of m^{7}G targets bound by IGF2BP3.

a Western blot of XRN2 in cell lysate from *in vitro* pulldown in HepG2 cells using antibodies that recognize IGF2BP1, IGF2BP2, or IGF2BP3. Rabbit IgG was used as a negative control. **b** Western blot of other exosome complex components in cell lysate from *in vitro* pulldown in HepG2 cells using antibodies that recognize IGF2BP1, IGF2BP2, or IGF2BP3. Rabbit IgG was used as a negative control. **c** mRNA levels (left) and protein levels (right) of EXOSC2 and IGF2BP3 upon *EXOSC2* knockdown and IGF2BP3 overexpression. Mean ± SEM of two independent experiments for mRNA expression level quantification. All western blots have been repeated independently twice with similar results. All source data are provided as a Source Data file.

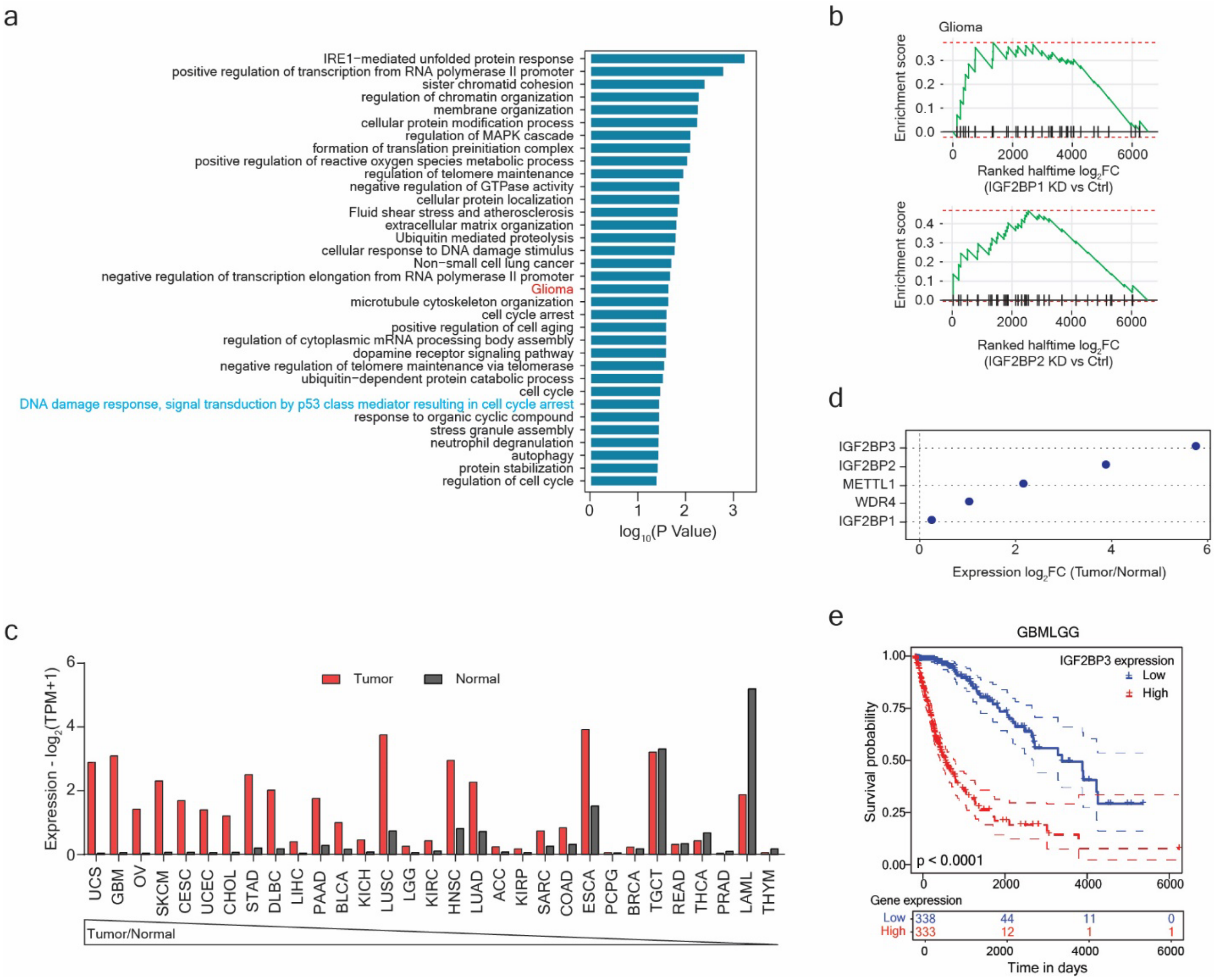

Supplementary Fig. 8: *IGF2BP3* is highly expressed in glioma.

a Functional enrichment analysis of genes that were bound by IGF2BPs and marked with m^7G. **b** Gene Set Enrichment Analysis (GSEA) of genes in 'Glioma' KEGG pathway against ranked list of genes according to half lifetime changes upon knocking down *IGF2BP1* (top) and *IGF2BP2* (bottom). **c** Kaplan-Meier survival analysis in TCGA database for glioma (TCGA-GBMLGG). The patients were divided into two groups of equal size based on *IGF2BP3* levels. *P* value was detected by the log-rank test. **d** Gene expression level $\log_2$FC comparing glioma with normal tissues using TCGA database for glioma (TCGA-GBMLGG). **e** Kaplan-Meier survival analysis in TCGA database for Glioma (TCGA-GBMLGG) with the entire dataset. The patients were divided into two groups of equal size based on *IGF2BP3* levels. *P* value was detected by the log-rank test.

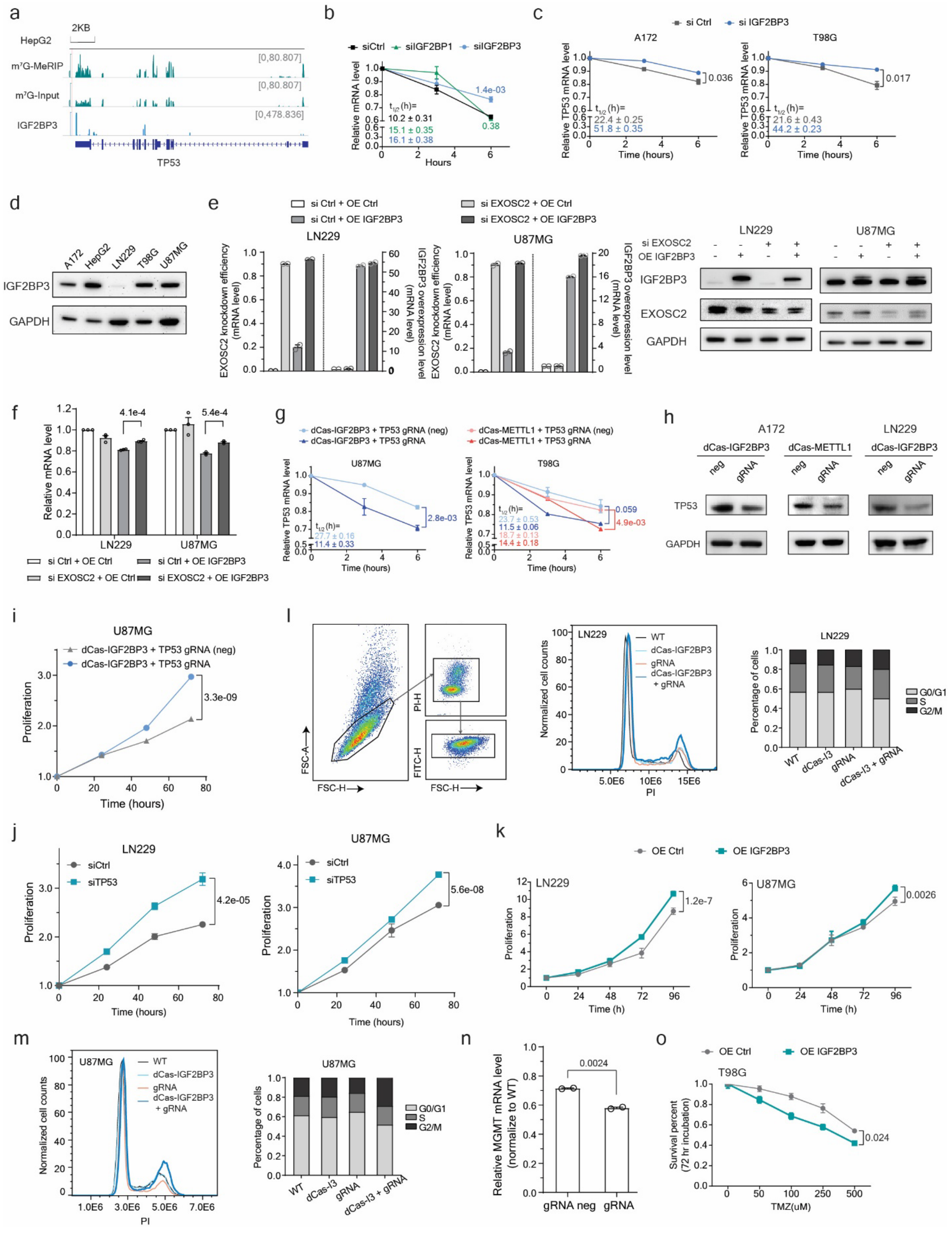

Supplementary Fig. 9: ***TP53*** **3'UTR m^{7}G methylation in cancer progression and chemoresistance**

a IGV plots showing the m^7G-MeRIP-seq peaks at the 3' UTR end of *TP53*, which is also overlapped with IGF2BP3 PAR-CLIP peaks. Y-axis showing counts per ten million reads. **b** Changes in *TP53* mRNA levels in HepG2 cells with *IGF2BP1* or *IGF2BP3* knockdown. Mean ± SEM of four independent experiments. Two-tailed Student's t-tests were used. *P* values are marked next to the dots. Calculated half lifetimes are marked in the corresponding colors. **c** Changes in *TP53* mRNA levels in lowly methylated cells upon *IGF2BP3* knockdown. Mean ± SEM of three independent experiments. Two-tailed Student's t-tests were used. *P* values are marked next to the dots. Calculated half lifetimes are marked in the corresponding colors. **d** Western blot showing relative quantity of IGF2BP3 proteins in cell lines. **e** mRNA levels (left) and protein levels (right) of EXOSC2 and IGF2BP3 upon *EXOSC2* knockdown and IGF2BP3 overexpression in LN229 and U87MG cells. Mean ± SEM of two independent experiments for mRNA expression level quantification. **f** mRNA levels of *TP53* upon IGF2BP3 overexpression, or *EXOSC2* knockdown, or both, in LN229 and U87MG cells. Mean ± SEM of three independent experiments. *P* values are marked on the top of each group. **g** Changes in mRNA levels in cells with introduction of dCas13b-IGF2BP3 or dCas13b-METTL1 and *TP53* guide RNA at the target locus or not (neg). Mean ± SEM of three independent experiments. Two-tailed Student's t-tests were used. *P* values are marked next to each group in the colors. Calculated half lifetimes are marked in the corresponding colors. **h** Western blot of TP53 protein levels in A172 and LN229 cells with dCas13b-IGF2BP3 or dCas13b-METTL1 tethering with the guide RNA and the negative gRNA. **i** Cell proliferation in U87MG cells using dCas13b-IGF2BP3 tethering with the guide RNA or the negative control gRNA. Mean ± SEM of six independent experiments, and two-tailed Student's t-tests were used. *P* values are marked next to the dots. **j** Cell proliferation in LN229 (left) and U87MG (right) cells with *TP53* knockdown by siRNA, compared to knockdown control. Mean ± SEM of six independent experiments, and two-tailed Student's t-tests were used. *P* values are marked next to the dots. **k** Cell proliferation in LN229 (left) and U87MG (right) cells with overexpression of IGF2BP3, compared to control. Mean ± SEM of four independent experiments, and two-tailed Student's t-tests were used. *P* values are marked next to the dots. **l-m** Distributions (left) and the normalized percentages (right) of the cells in the G0/G1, S, and G2/M phases in LN229 (**l**) and U87MG (**m**) cells. The very left in **l** presents the gating strategy of the cell cycle assay. **n** Relative MGMT mRNA levels in T98G cells with dCas13b-IGF2BP3 tethering with the guide RNA or negative gRNA. Mean ± SEM of two independent experiments, and two-tailed Student's t-tests were used. **o** Survival percentages of the T98G cells with IGF2BP3 overexpression compared to control treated with different concentrations of TMZ. Mean ± SEM of four independent experiments, and two-tailed Student's t-tests were used. *P* values are marked next to the dots. All western blots have been repeated independently twice with similar results. All source data are provided as a Source Data file.

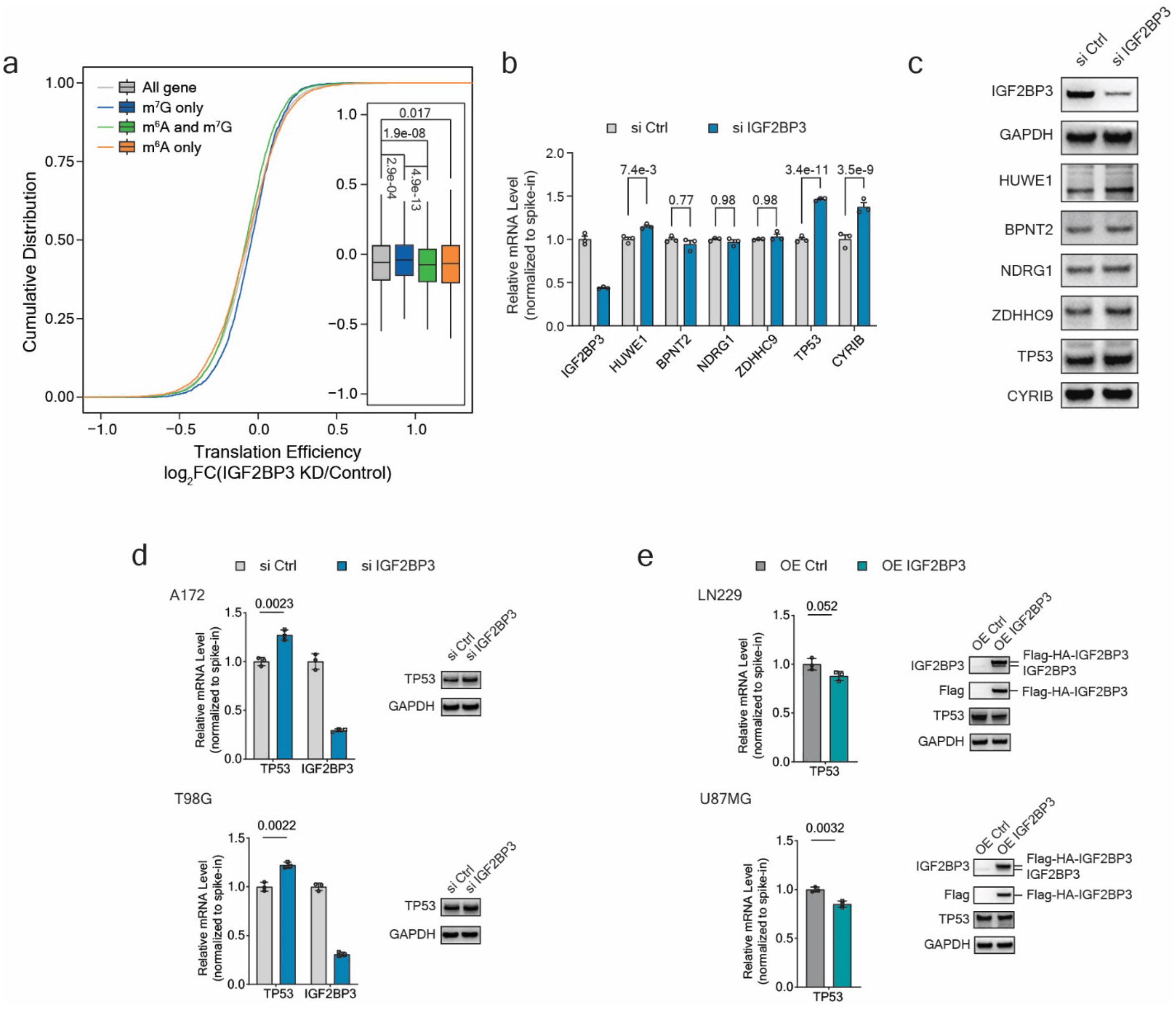

Supplementary Fig. 10: IGF2BP3 promotes degradation of m^{7}G targets and affects protein levels.

a Cumulative curve of log$_2$FC (fold changes) distribution of translation efficiency (ratio of ribosome bound fragments to mRNA input) between si*IGF2BP3* and siControl transfection in HepG2 cells, with groups of all gene (gray), targets with only m^{7}G modification (blue), targets with only m^{6}A modification (orange), and targets with both modifications (green). IGF2BP3 promoted translation of m^{6}A targets but showed a complicated but mild effect on m^{7}G targets with a slight inhibition on their translation. **b** RT-qPCR results of relative mRNA expression level of the representative m^{7}G-modified targets upon *IGF2BP3* knockdown in HepG2 cells. *IGF2BP3* knockdown not only stabilized all related transcripts but also led to increased mRNA levels of HUWE1, CYRIB, and TP53, with little effect on other targets. Mean ± SEM of three independent experiments. Two-tailed Student's t-tests were used. *P* values are marked at the top of each group. **c** Western blotting results of relative protein expression level of the representative m^{7}G-modified targets upon *IGF2BP3* knockdown in HepG2 cells. HUWE1 and TP53 showed increases in protein expression upon *IGF2BP3* knockdown. **d** RT-qPCR results of relative TP53 mRNA expression level (left) and western blotting results of relative TP53 protein expression level (right) upon *IGF2BP3* knockdown in the cell lines with highly methylated TP53 transcripts. *IGF2BP3* knockdown induced increases in both mRNA and protein expression levels of TP53. Mean ± SEM of three independent experiments for RT-qPCR. Two-tailed Student's t-tests were used. *P* values are marked at the top of each group. **e** RT-qPCR results of

relative TP53 mRNA expression level (left) and western blotting results of relative TP53 protein expression level (right) upon IGF2BP3 overexpression in the cell lines with lowly methylated TP53 transcripts. IGF2BP3 overexpression induced decreases in both mRNA and protein expression levels of TP53. Mean ± SEM of three independent experiments for RT-qPCR. Two-tailed Student's t-tests were used. *P* values are marked at the top of each group. Western blotting results also confirmed the overexpression of IGF2BP3 with Flag tag. All source data are provided as a Source Data file.
