# Supplementary material for: IGF2BP3 promotes mRNA degradation through internal m7G modification
Source: Nat Commun. 2024 Aug 28;15:7421. doi: 10.1038/s41467-024-51634-w (PMC11358264; doi:10.1038/s41467-024-51634-w)
Supplement: Supplementary file 3 — Description of Additional Supplementary Information [file 41467_2024_51634_MOESM3_ESM.docx]

**Description of Additional Supplementary Files**

File Name: Supplementary Data 1

Description: Top proteins enriched by m7G probes based on the proteomics data

File Name: Supplementary Data 2

Description: Primers for dCas13b construction, guide RNA target sequences, and qPCR primers used in this study
