# Supplementary material for: IGF2BP3 promotes mRNA degradation through internal m7G modification
Source: Nat Commun. 2024 Aug 28;15:7421. doi: 10.1038/s41467-024-51634-w (PMC11358264; doi:10.1038/s41467-024-51634-w)
Supplement: Supplementary file 6 — Reporting Summary [file 41467_2024_51634_MOESM6_ESM.pdf]

nature portfolio

Corresponding author(s): Chuan He

Last updated by author(s): 07/09/2024

Reporting Summary

Nature Portfolio wishes to improve the reproducibility of the work that we publish. This form provides structure for consistency and transparency in reporting. For further information on Nature Portfolio policies, see our Editorial Policies and the Editorial Policy Checklist.

Statistics

For all statistical analyses, confirm that the following items are present in the figure legend, table legend, main text, or Methods section.

n/a	Confirmed	
☐	☒	The exact sample size (*n*) for each experimental group/condition, given as a discrete number and unit of measurement
☐	☒	A statement on whether measurements were taken from distinct samples or whether the same sample was measured repeatedly
☐	☒	The statistical test(s) used AND whether they are one- or two-sided<br>*Only common tests should be described solely by name; describe more complex techniques in the Methods section.*
☒	☐	A description of all covariates tested
☒	☐	A description of any assumptions or corrections, such as tests of normality and adjustment for multiple comparisons
☐	☒	A full description of the statistical parameters including central tendency (e.g. means) or other basic estimates (e.g. regression coefficient) AND variation (e.g. standard deviation) or associated estimates of uncertainty (e.g. confidence intervals)
☐	☒	For null hypothesis testing, the test statistic (e.g. *F*, *t*, *r*) with confidence intervals, effect sizes, degrees of freedom and *P* value noted<br>*Give P values as exact values whenever suitable.*
☒	☐	For Bayesian analysis, information on the choice of priors and Markov chain Monte Carlo settings
☒	☐	For hierarchical and complex designs, identification of the appropriate level for tests and full reporting of outcomes
☐	☒	Estimates of effect sizes (e.g. Cohen's *d*, Pearson's *r*), indicating how they were calculated

Our web collection on statistics for biologists contains articles on many of the points above.

Software and code

Policy information about availability of computer code

Data collection	Illumina NovaSeq 6000 System
Data analysis	Trimmomatic (version 0.39), FastQC (version 0.11.8), Cutadapt (version 2.10), Hisat2 (version 2.1.0), Bowtie2 (version 2.4.1), STAR (version 2.7.3a), Samtools (version 1.9), HTSeq (version 0.12.4), Homer (version 4.11), MACS2 (version 2.2.7.1), Bedtools (version 2.26), DAVID 6.8 (Oct. 2016), ImageJ (version 1.49p), IGV (v2.13.1), featureCounts (version 2.0.6), DESeq2 (version 1.44.0).

For manuscripts utilizing custom algorithms or software that are central to the research but not yet described in published literature, software must be made available to editors and reviewers. We strongly encourage code deposition in a community repository (e.g. GitHub). See the Nature Portfolio guidelines for submitting code & software for further information.

Data

Policy information about availability of data

All manuscripts must include a data availability statement. This statement should provide the following information, where applicable:

- Accession codes, unique identifiers, or web links for publicly available datasets
- A description of any restrictions on data availability
- For clinical datasets or third party data, please ensure that the statement adheres to our policy

m7G-MeRIP-seq in WT HepG2 cells, knockdown control cells, and METTL1 stable knockdown HepG2 cells, and m7G-seq in HepG2 cells were downloaded and re-analyzed in this study (GSE112276). The sequencing data produced in this study have been deposited in Gene Expression Ominibus (GEO) repository under the accession number GSE241222. The mass spectrometry proteomics data have been deposited to the ProteomeXchange Consortium via the PRIDE75 partner

repository with the dataset identifier PXD049390 and 10.6019/PXD049390.

Human research participants

Policy information about studies involving human research participants and Sex and Gender in Research.

Reporting on sex and gender	N/A. This work does not involve human research participants.
Population characteristics	N/A. This work does not involve human research participants.
Recruitment	N/A. This work does not involve human research participants.
Ethics oversight	N/A. This work does not involve human research participants.

Note that full information on the approval of the study protocol must also be provided in the manuscript.

Field-specific reporting

Please select the one below that is the best fit for your research. If you are not sure, read the appropriate sections before making your selection.

[x] Life sciences [ ] Behavioural & social sciences [ ] Ecological, evolutionary & environmental sciences

For a reference copy of the document with all sections, see nature.com/documents/nr-reporting-summary-flat.pdf

Life sciences study design

All studies must disclose on these points even when the disclosure is negative.

Sample size	No statistical methods were used to predetermine sample size. Sample size were determined based on our prior experience on similar experiments and literature reports. For cell-based assays, samples were collected till we have sufficient number to obtain reliable statistics.
Data exclusions	No data were excluded.
Replication	Results were confirmed in at least two biological replicates for each experiment as stated in the figure legends. All attempts to replicate data are successful.
Randomization	The experiments were not randomized. Controlling for covariates was unnecessary because all assays were performed in pairs.
Blinding	Blinding was not applicable because the focus of this paper did not involve group allocation and blinding.

Reporting for specific materials, systems and methods

We require information from authors about some types of materials, experimental systems and methods used in many studies. Here, indicate whether each material, system or method listed is relevant to your study. If you are not sure if a list item applies to your research, read the appropriate section before selecting a response.

Materials & experimental systems

n/a	Involved in the study	
[ ]	[x]	Antibodies
[ ]	[x]	Eukaryotic cell lines
[x]	[ ]	Palaeontology and archaeology
[x]	[ ]	Animals and other organisms
[x]	[ ]	Clinical data
[x]	[ ]	Dual use research of concern

Methods

n/a	Involved in the study	
[x]	[ ]	ChIP-seq
[ ]	[x]	Flow cytometry
[x]	[ ]	MRI-based neuroimaging

Antibodies

Antibodies used	Mouse anti-m7G (RN017M; MBL, clone 4141-13). Rabbit anti-METTL1 (14994-1-AP; Proteintech). Rabbit anti-m6A (E1610S; NEB). Mouse anti-WDR4 (sc-100894; Santa Cruz). Rabbit anti-IGF2BP1 (8482; Cell Signaling). Rabbit anti-IGF2BP2 (14672; Cell Signaling). Rabbit anti-IGF2BP3 (57145; Cell Signaling). Rabbit anti-EXOSC2 (ab181211; Abcam). Mouse anti-EXOSC3 (sc-166568; Santa Cruz). Mouse anti-EXOSC4 (sc-166772; Santa Cruz). Mouse anti-EXOSC7 (sc-393686; Santa Cruz). Rabbit anti-XRN2 (13760; Cell Signaling). Mouse anti-p53 (sc-126; Santa Cruz). Mouse anti-MGMT (sc-166528; Santa Cruz). Mouse Anti-BrdU (B2531; Sigma; clone BU-33). Goat Anti-Mouse IgG H&L (Alexa Fluor® 488) (ab150113; Abcam). Goat anti-rabbit IgG-HRP (7074; Cell Signaling). Horse anti-mouse

IgG-HRP (7076; Cell Signaling). Rabbit anti-GAPDH-HRP (8884; Cell Signaling).

Validation

IGF2BP1: IP validated in our hand. Manufactures: Rabbit monoclonal, applications in WB, IP
IGF2BP2: IP validated in our hand. Manufactures: Rabbit monoclonal, applications in WB
IGF2BP3: IP validated in our hand. Manufactures: Rabbit monoclonal, applications in WB
METTL1: IP validated in our hand. Manufactures: Rabbit polyclonal, KO validated, applications in WB, IP, IHC, IF, ELISA
WDR4: IP validated in our hand. Manufactures: Mouse monoclonal, applications in WB, IP, IF, IHC(P) and ELISA
Antibodies were validated by the manufacturers and used in accordance with the manufacturers recommendations.

Eukaryotic cell lines

Policy information about cell lines and Sex and Gender in Research

Cell line source(s)

HepG2 cells are purchased from ATCC (catalog No: HB-8065).
A172 cells are purchased from ATCC (catalog No: CRL-1620).
LN229 cells are purchased from ATCC (catalog No: CRL-2611).
T98G cells are purchased from ATCC (catalog No: CRL-1690).
U87MG cells are purchased from ATCC (catalog No: HTB-14).

Authentication

Cell lines were not authenticated after purchase from ATCC.

Mycoplasma contamination

All cell lines used in this study were tested negative of mycoplasma contamination.

Commonly misidentified lines
(See ICLAC register)

No commonly misidentified line was used.

Flow Cytometry

Plots

Confirm that:

- [x] The axis labels state the marker and fluorochrome used (e.g. CD4-FITC).
- [x] The axis scales are clearly visible. Include numbers along axes only for bottom left plot of group (a 'group' is an analysis of identical markers).
- [x] All plots are contour plots with outliers or pseudocolor plots.
- [x] A numerical value for number of cells or percentage (with statistics) is provided.

Methodology

Sample preparation

LN229 and U87MG cells were labeled with BrdU (10 µM) for 30 minutes under normal incubator conditions, then trypsinized and fixed with 70% ethanol overnight. After fixation, cell pellets were collected, resuspended in 200 µL 4M HCl, and incubated at room temperature for 20 minutes, followed by neutralization with Borax. The cell pellets were washed twice with 1% BSA in PBS before incubation with the BrdU primary antibody (B2531; Sigma; clone BU-33) at room temperature for 30 minutes in the dark. After three washes, the pellets were incubated with goat anti-mouse IgG Alexa Fluor 488 secondary antibody (ab150113; Abcam) at room temperature in the dark for 1 hour. Cell pellets were then washed three times with 1 mL 0.1% Triton X-100 and 1% BSA in PBS before incubation with RNase A and propidium iodide (PI) at a final concentration of 5 µg/mL PI for 15-30 minutes at room temperature in the dark.

Instrument

BD LSR Fortessa flow cytometer

Software

FlowJo software (BD Biosciences) v10

Cell population abundance

About 50-60% cells were used in the final quantification. The gating of single cells excluded about 40-50% of the signals and the following gating for PI and FITC signals mostly included all the cells. Cells at different cell cycle stages were further quantified with Cell Cycle model in Flowjo.

Gating strategy

Single cells gating (FSC-H vs A) followed by the gating confirmation with PI and FITC channels.

- [x] Tick this box to confirm that a figure exemplifying the gating strategy is provided in the Supplementary Information.
